# The protective effect of the Vacha rhizome extract on chronic stress-induced immunodeficiency in rat

**DOI:** 10.1080/13880209.2017.1301495

**Published:** 2017-03-17

**Authors:** H. N. Sarjan, S. Divyashree, H. N. Yajurvedi

**Affiliations:** Department of Zoology, University of Mysore, Manasagangotri, Mysore, India

**Keywords:** Apoptosis, forced swimming, leukocytes, lymphoid organs

## Abstract

**Context:** Chronic stress is an inevitable factor in the modern day society which affects cell mediated as well as humoral immunity. There is a need to prevent stress effects with traditionally used herbs.

**Objective:** The present study was undertaken to investigate the immunoprotective effect of Vacha (*Acorus calamus* L. Acoraceae) rhizome under stressful condition.

**Materials and methods:** Soxlet extraction of Vacha rhizome was performed with increasing polarity of solvents, i.e., petroleum ether to ethanol. The extract was concentrated by distilling off the solvent in flash evaporator and dried in desiccators. The benzene extract was found to have anti-stress property in our earlier studies and hence it was used in the present experiment. Extract was administered every day for 4** **weeks orally to adult female rats prior to exposure to stress, restraint (1** **h) and forced swimming exercise (15** **min).

**Results:** Vacha rhizome extract significantly prevented the stress induced reduction in total and differential leukocytes count, immunoglobulin content, bone marrow cellularity and viability, lymphocytes counts in lymphoid organs, islands of white pulp of spleen (ED_50_ = 10 mg, *p* < 0.001) and a significant increase in circulating immune complexes and apoptotic index of lymphoid organs (ED_50_ = 10 mg, *p* < 0.001) compared to controls.

**Discussion and conclusion:** The present study clearly indicates that Vacha extract not only prevents stress-induced suppression of immunity and structural involution of lymphoid organs, but also boosts immunity in normal rats. Therefore, it is suggested that Vacha extract administration maintains normal immunity despite the body experiencing stress.

## Introduction

Stress elicits different physiological and behavioral changes in the body mediated by the activation of the HPA axis and the SNS axis (Padgett & Glaser 2003). Stress is involved in the etiopathogenesis of a variety of disorders, including immunosuppression (Shi et al. 2003), diabetes mellitus (Pouwer et al. 2010), sexual dysfunction (Geraghty et al. 2015), peptic ulcer (Levenstein et al. 1999), hypertension (Spruill 2010), etc. The immune system involves organized and well-regulated system of cells and molecules with specialized roles in defense (Kumari et al. 2014). Any disturbance in the immune functions leads to the development of immune disorders. Chronic stress is one of such factors which affects both cell mediated and humoral immunity (Padgett & Glaser 2003) and markedly affects immunological parameters viz. leukocyte count (Moazzam et al. 2012), leukocyte subset distribution (Coe & Lubach 2005), cytokine production (Agarwal & Marshall 1998), immunoglobulins (Archana & Namasivayam 2000), natural killer cell activity (Li et al. 2012) and macrophage maturation and activity (Quinteiro-Filho et al. 2010) in humans and animals. Stress cannot be avoided in the present competitive world. Hence, in view of the health implications of chronic stress, studies are needed to prevent stress effects despite undergoing stressful experiences.

There is a great preference for the usage of herbs for immunoprotection as synthetic drugs have adverse effects (Rachh et al. 2014). Plant-based drugs are more effective to use as an immune strengthener in normal individual as well as under diseased conditions (Ravichandiran & Vishal 2015). Several Indian medicinal plants are extensively used in traditional medicine for various disorders. Several studies in animal models focused on immune-enhancing property of different herbal extracts viz. *Eclipta protrata* L. (Asteraceae) (Karthikumar et al. 2011), *Celastrus paniculatus* L. (Celastraceae) (Salomi et al. 2011), *Withania somnifera* L. (Solanaceae) (Verma et al. 2012), *Prunus cerasus* L. (Rosaceae) (Ali et al. 2013) and *Garcinia cambogia* L. (Clusiaceae) (Goudarzvand et al. 2016). Similarly Kombucha tea amelioratesd the autoimmune encephalomyelitis in mouse model of multiple sclerosis (Marzban et al. 2015). Few herbs viz. *Withania somnifera* (Bhattacharyaa & Muruganandam 2003), *Spirullina platensis* L. (Phormidiaceae) (Juvekar & Nachankar 2005) and *Habenaria intermedia* D. Don. (Orchidacea) (Habbu et al. 2012) provide immunoprotection under stressful conditions in mice and rats. Hence, immunostimulatory plants are likely candidates to maintain a disease-free state and can be helpful in therapy. Therefore, there is a need for more investigations on effective and safer immunomodulatory and immunoprotective herbal products to lessen the stress effects on the immune system. The herbal extracts investigated for immunoprotection (Bhattacharyaa & Muruganandam 2003; Juvekar & Nachankar 2005; Habbu et al. 2012) under stressful conditions mainly focused on blood leukocytes and phagocytosis. However, studies involving different parameters viz. humoral immunity, bone marrow cellularity, and alterations in different lymphoid organs, are necessary to better understand the effectiveness of herbal extracts. In addition, the minimum dosage of the extracts used to prevent stress-induced immune dysfunctions was 25 mg/kg body weight. Further, thus far only two studies have been reported on immunomodulation and immunoprotection effects of *Acorus calamus* (Vacha). In an *in vitro* study, petroleum ether, alcohol and volatile oil extracts of Vacha leaves stimulated the phagocytosis in human neutrophils (Ravichandiran & Vishal 2015). Ethyl acetate extract of Vacha rhizome and α-asarone administration significantly prevented the depletion of CD4 T, CD8 T, IL-2, IFN-γ and enhanced IL-4 levels in rats exposed to noise (Dharini et al. 2012). Though these studies reveal immunomodulatory property of Vacha, a comprehensive study involving a variety of immunological parameters as mentioned above is needed to understand immunomodulatory properties of Vacha. Therefore, the present study aims to investigate whether rhizome extract of Vacha ameliorates stress-induced immunological alterations and also boosts immunity in unstressed rats.

## Materials and methods

### Animals

Adult female Wistar rats weighing 180–200 g were obtained from the Central Animal Facility, University of Mysore, Mysore. The rats were provided standard rat chow and water *ad libitum* and were kept in 27 ± 2 °C, under 12 h light/dark cycle (lights on 07:00–19:00 h) in polypropylene cages. All procedures performed in the studies involving animal participants were in accordance with the ethical standards of the Committee for the Purpose of Control and Supervision of Experiments on Animals (CPCSEA), India. Approval for the proposed animal experiments was obtained from the Institutional Animal Ethics Committee of University of Mysore, India (Reference number – UOM/IAEC/17/2013, dated 28/09/2013).

### Procedure for inducing stress

Two kinds of stressors were used (Grissom et al. 2008).

- ***Restraint:***A rat was placed in an open-ended glass cylindrical restrainer (6.7 cm in diameter and 22.3 cm in length) for 1 h.
- ***Forced swimming exercise***: Rats were individually forced to swim for 15 min in a chromatography jar (18″ high ×8.75″ outer diameter) filled 2/3 full of water at room temperature 27 ± 2 °C.

### Plant and preparation of plant extract

*A. calamus* was collected from Mysore Ayurvedic Medical College Park, Mysore, Karnataka, India during the month of February 2015 and was authenticated by a Botanist, Dr. S. Mahadevakumar, Department of Studies in Botany, Univeristy of Mysore, India (Voucher specimen accession no. 160). The rhizome of the *A. calamus* was shade dried and a coarse powder was prepared. The powder was extracted at room temperature in a Soxhlet apparatus with benzene. The extract was concentrated by distilling off the solvent in flash evaporator and dried in desiccators.

### Experimental protocol

Rats of similar age were randomly divided into 5 groups, each group consisting of 5 animals.

Group I (control): The rats were maintained without any disturbance.

Group II (vehicle control): Each rat was administered orally 0.5 mL of 1% carboxy methyl cellulose.

Group III (unstressed + Vacha extract): The rats were administered orally benzene extract of Vacha (10 mg/kg bw/0.5 mL/rat) orally every day for 4 weeks.

Group IV (stress): The rats were exposed to stress regime, i.e., restraint followed by forced swimming daily for 4 weeks.

Group V (stress + Vacha extract): The rats were administered orally the crude benzene extract of Vacha (10 mg/kg bw/0.5 mL/rat) and 1 h later exposed to stressors similar to those in group IV.

Initial body weight of all the animals was recorded before the commencement of the experiment. The rats were killed, 24 h after last treatment. At autopsy, rats were weighed and for hematological studies blood was drawn by heart puncture. The blood was centrifuged at 2500 rpm for 10 min and serum was stored at −20 °C for estimation of total immunoglobulin and circulating immune complexes. The lymphoid organs (spleen, thymus, and axillary lymph nodes) were removed, cleared of fat tissues, weighed and stored (−20 °C) until apoptosis assays were conducted. The absolute organ weights were converted into relative organ weight (weight/100 g body weight).

### Immunological parameters

#### Haematological procedure

Total leukocyte count was conducted using Neubauer counting chamber after diluting the blood with WBC diluting fluid. Total leukocyte count was expressed per microlitre of blood. Differential leukocyte count, i.e., percentage of lymphocytes, monocytes, neutrophils, basophils and eosinophils was carried out by using Giemsa stained blood smear.

### Estimation of total immunoglobulin content

The total immunoglobulins level was estimated by zinc sulfate turbidity test (Ibrahim 2014). The serum sample (25 μL) mixed with 1.7 mL of 0.7 mm, zinc sulfate (pH 5.8) served as sample tube and serum mixed with phosphate buffered saline at the same ratio was used as control tube. Both the mixtures were shaken and allowed to stand at room temperature for 1 h. The turbidity was measured at 545 nm and expressed as ZST unit and the ZST unit was finally converted into mg/mL.

### Estimation of circulating immune complexes

Concentration of circulating immune complexes was estimated by polyethylene glycol precipitation method (Haskova et al. 1978). Two mL of 0.1 M borate buffer (pH 8.4) and 2 mL of 4.16% buffered polyethylene glycol (in borate buffer) were separately added to 0.22 mL of serum. Serum was pre-diluted 1:3 with borate buffer. The solutions were mixed and incubated at room temperature for 60 min and absorbance was measured at 450 nm. The PEG index was calculated by using the formula,

PEG index = (Absorbance with polyethylene glycol − Absorbance with Borate buffered saline) × 1000

### Isolation of bone marrow stem cells and viability test

Bone marrow stem cells were isolated from the femur of the hind leg, according to the procedure of Madaan et al. (2014) with some modifications. Briefly, hind leg was excised from the animal and femur was removed. Bone marrow was flushed out of the femur with 1 mL of sterile HBSS solution. The cell suspension was centrifuged at 2000 rpm at 4 °C for 10 min. RBCs were lysed by using the 10X RBC lysing solution. The harvested cells were counted using Neubauer counting chamber and their viability was assessed by trypan blue exclusion method using 0.2% trypan blue in phosphate buffered saline.

### Lymphocyte counts in lymphoid organs

Lymphocytes were isolated from spleen, thymus and axillary lymph nodes by pressing them through 400 μm sterile nylon mesh. The cell suspension was centrifuged at 2000 rpm at 4 °C for 5 min and the cell pellet was collected. The cell pellet was washed and suspended in the HBSS medium and counted in a Neubauer counting chamber with the help of Giemsa stain.

### Apoptosis

Spleenocytes, thymocytes and lymphocytes of axillary lymph nodes were isolated by pressing the organs through 400 μm sterile nylon mesh using phosphate buffered saline. The cell suspension was centrifuged at 2000 rpm at 4 °C for 5 min and the cell pellet was collected. Ten microlitres each of ethidium bromide (100 μg/mL), acridine orange (100 μg/mL) and cell pellet were mixed and loaded in the Neubauer counting chamber. Cells were observed under fluorescent microscope using 450 nm and 530 nm filters, where, healthy cells appeared green and apoptotic cells as red. Apoptotic index (%) was calculated by dividing the number of apoptotic cells by the total number of cells counted and multiplied with 100 (Grossmann et al. 1998).

### Histological examination

Paraffin blocks and 5 μm thick sections of the spleen, thymus and axillary lymph node were prepared. The sections were stained with hematoxylin and eosin for histological observations. In the spleen, number of islands of white pulp was counted from 50 randomly selected cross sections.

### Estimation of adrenal steroid dehydrogenase activity

The activity of key steroidogenic enzyme 3β-hydroxysteroid dehydrogenase (3β-HSDH) was estimated in the adrenal gland, according to procedure of Shivanandappa and Venkatesh (1997).

### Statistical analysis

The mean ± SEM of each parameter was computed considering the data on at least 5 rats per group and mean values of each parameter of different groups were compared using one way ANOVA followed by Duncan's multiple range test and judged significant if *p* < 0.05.

## Results

### Weight of the body and lymphoid organs

The body weight and relative weight of spleen, thymus and axillary lymph nodes showed a significant decrease in stressed rats compared to controls where as those of Vacha extract pretreated stressed rats did not differ from controls. There was a significant increase in the relative weight of lymphoid organs in Vacha extract treated unstressed rats compared to the controls, whereas the vehicle treated rats did not significantly differ from controls (Table 1).

**Table 1.** Effects of Vacha rhizome extract on body and lymphoid organ weight of rats.

Groups and treatments	% Change in body weight (g) ± SE	Weight (mg)/100 g body weight mean ± SE		
		Spleen	Thymus	Axillary lymph nodes
Control	13.653 ± 2.276^a^	287.666 ± 3.480^b^	41.666 ± 3.282^b^	48.333 ± 4.910^b^
Vehicle control	12.081 ± 0.822^a^	283.750 ± 8.910^b^	39.000 ± 3.949^b^	49.333 ± 1.452^b^
Unstressed + benzene extract of Vacha (10 mg/kg body weight)	15.471 ± 1.144^a^	310.000 ± 5.773^a^	56.333 ± 4.841^a^	61.000 ± 1.732^a^
Stress	7.289 ± 0.895^b^	255.666 ± 6.565^c^	24.500 ± 1.936^c^	36.333 ± 2.603^c^
Stress + benzene extract of Vacha (10 mg/kg body weight)	13.707 ± 0.608^a^	286.800 ± 4.115^b^	41.400 ± 2.379^b^	47.750 ± 3.794^b^
ANOVA F Value (df =4, 20)	7.104 *p* < 0.001	8.023 *p* < 0.001	9.896 *p* < 0.001	6.489 *p* < 0.001

Mean values with same superscript letters in the given column are not significantly different, whereas those with different superscript letters are significantly (*p* < 0.05) different as judged by Duncan’s multiple test, df: degree of freedom.

### Weight of adrenal gland and adrenal 3β-HSDH activity

There was a significant increase in the relative weight of the adrenal gland and adrenal 3β-HSDH activity in stressed rats compared to controls, whereas those of Vacha extract pretreated stressed rats did not differ from controls (Table 2).

**Table 2.** Effects of Vacha rhizome extract on adrenal 3β-HSDH activity and weight of the adrenal gland of rats.

Groups and treatments	Adrenal 3β-HSDH activity (nmol/mg/min) ± SE	The relative weight of the adrenal gland (mg/100 g of body weight) ± SE
Control	0.452 ± 0.013^a^	20 ± 0.001^a^
Vehicle Control	0.447 ± 0.031^a^	19 ± 0.000^a^
Unstressed + benzene extract of Vacha (10 mg/kg body weight)	0.456 ± 0.023^a^	19 ± 0.000^a^
Stress	0.583 ± 0.044^b^	26 ± 0.001^b^
Stress + benzene extract of Vacha (10 mg/kg body weight)	0.455 ± 0.028^a^	19 ± 0.001^a^
ANOVA F Value (df =4, 20)	3.571 *p* < 0.01	4.159 *p* < 0.01

Mean values with same superscript letters in the given column are not significantly different, whereas those with different superscript letters are significantly (*p* < 0.05) different as judged by Duncan’s multiple test, df: degree of freedom.

### Haematological studies

Total leukocyte count and counts of lymphocytes, neutrophils, monocytes, basophils and eosinophils showed a significant decrease in stress group rats compared to controls and those of Vacha extract pretreated stressed rats. The total leukocyte count and differential leukocyte count of Vacha extract treated unstressed rats were significantly higher than controls (Table 3).

**Table 3.** Effects of Vacha rhizome extract on total and differential counts of leukocytes of rats.

Groups and Treatments	Mean total count/μL of leukocytes ± SE	Mean count/μL ± SE				
		Lymphocytes	Neutrophils	Monocytes	Basophils	Eosinophils
Control	4882.000 ± 138.108^b^	4168.200 ± 95.876^b^	524.250 ± 27.756^b^	165.750 ± 15.611^b^	65.400 ± 6.523^b^	48.500 ± 1.190^b^
Vehicle Control	4844.000 ± 267.536^b^	3895.200 ± 260.199^b^	540.000 ± 21.122^b^	169.600 ± 24.254^b^	69.500 ± 5.041^b^	48.000 ± 3.082^b^
Unstressed + benzene extract of Vacha (10 mg/kg body weight)	5730.000 ± 320.208^a^	4832.333 ± 269.499^a^	662.000 ± 32.254^a^	230.000 ± 15.038^a^	96.500 ± 16.500^a^	71.000 ± 4.020^a^
Stress	3146.000 ± 299.359^c^	2512.600 ± 247.264^c^	311.250 ± 66.683^c^	62.333 ± 2.027^c^	35.750 ± 5.022^c^	22.750 ± 4.571^c^
Stress + benzene extract of Vacha (10 mg/kg body weight)	4176.666 ± 186.755^b^	3591.000 ± 132.061^b^	489.250 ± 29.329^b^	161.250 ± 8.330^b^	69.250 ± 3.520^b^	45.250 ± 3.567^b^
ANOVA F Value (df =4, 20)	13.844 *p* < 0.001	15.536 *p* < 0.001	9.536 *p* < 0.001	11.638 *p* < 0.001	8.338 *p* < 0.001	22.538 *p* < 0.001

Mean values with same superscript letters in the given column are not significantly different, whereas those with different superscript letters are significantly (*p* < 0.05) different as judged by Duncan’s multiple test, df: degree of freedom.

### Total immunoglobulin content and circulating immune complexes

The total immunoglobulin contents in stressed rats were significantly lower than controls, but not in Vacha extract pretreated stressed rats, whereas it was significantly higher than controls in Vacha extract treated unstressed rats. Circulating immune complexes showed a significant increase in stressed rats compared to controls and that of Vacha extract pretreated stressed rats did not differ from controls. In Vacha extract treated unstressed rats, the content of circulating immune complexes was significantly lower than controls (Table 4).

**Table 4.** Effects of Vacha rhizome extract on circulating immune complexes and total immunoglobulins contents of rats.

Groups and treatments	Total immunoglobulins Content (mg/mL) ± SE	Circulating immune complexes PEG index ± SE
Control	0.542 ± 0.009^b^	78.600 ± 2.249^b^
Vehicle Control	0.537 ± 0.044^b^	78.000 ± 2.915^b^
Unstressed + benzene extract of Vacha (10 mg/kg body weight)	0.645 ± 0.045^a^	44.200 ± 2.745^c^
Stress	0.372 ± 0.023^c^	126.000 ± 2.983^a^
Stress + benzene extract of Vacha (10 mg/kg body weight)	0.499 ± 0.028^b^	75.800 ± 3.382^b^
ANOVA F Value (df = 4, 20)	10.389 *p* < 0.001	113.690 *p* < 0.001

Mean values with same superscript letters in the given column are not significantly different, whereas those with different superscript letters are significantly (*p* < 0.05) different as judged by Duncan’s multiple test, df: degree of freedom.

### The number of bone marrow stem cells and their viability

There was a significant decrease in the number of bone marrow stem cells as well as their viability as is shown by an increase in the % of trypan blue stained cells in stressed rats compared to controls. However, these in Vacha extract pretreated stressed rats did not differ from controls, whereas Vacha extract treated unstressed rats showed a significant increase in the number of bone marrow stem cells compared to controls (Table 5).

**Table 5.** Effects of Vacha rhizome extract on bone marrow stem cells of rats.

Groups and treatments	The number of bone marrow stem cells (×10^4^/mL) ± SE	Viability of bone marrow stem cells (%) ± SE
Control	773.715 ± 8.699^b^	96.200 ± 0.860^a^
Vehicle control	753.124 ± 7.270^b^	93.800 ± 0.860^a^
Unstressed + benzene extract of Vacha (10 mg/kg body weight)	853.590 ± 48.451^a^	96.000 ± 1.000^a^
Stress	664.862 ± 17.344^c^	86.800 ± 1.392^b^
Stress + benzene extract of Vacha (10 mg/kg body weight)	738.906 ± 14.835^b^	93.000 ± 0.707^a^
ANOVA F Value (df = 4, 20)	8.713 *p* < 0.001	14.027 *p* < 0.001

Mean values with same superscript letters in the given column are not significantly different, whereas those with different superscript letters are significantly (*p* < 0.05) different as judged by Duncan’s multiple test, df: degree of freedom.

### Lymphocyte counts in lymphoid organs

The number of lymphocytes were significantly decreased in spleen, thymus and axillary lymph node of stressed rats compared to controls and those of Vacha extract pretreated stressed rats did not differ from controls. Lymphocyte count was significantly increased in all the lymphoid organs of Vacha extract treated unstressed rats (Table 6).

**Table 6.** Effects of Vacha rhizome extract on lymphocyte counts in different lymphoid organs of rats.

Groups and treatments	The number of lymphocytes (× 10^6^/mL) ± SE		
	Spleen	Thymus	Axillary lymph nodes
Control	188.00 ± 2.213^b^	1530.400 ± 2.227^b^	754.200 ± 3.624^b^
Vehicle control	179.000 ± 5.431^b^	1527.000 ± 3.114^b^	743.400 ± 1.964^b^
Unstressed + benzene extract of Vacha (10 mg/kg body weight)	229.800 ± 12.276^a^	1724.400 ± 11.732^a^	799.800 ± 7.009^a^
Stress	142.000 ± 16.077^c^	1405.400 ± 6.874^c^	545.600 ± 25.085^c^
Stress + benzene extract of Vacha (10 mg/kg body weight)	174.000 ± 2.645^b^	1510.333 ± 2.603^b^	726.200 ± 3.611^b^
ANOVA F Value (df =4, 20)	44.532 *p* < 0.001	281.433 *p* < 0.001	67.900 *p* < 0.001

Mean values with same superscript letters in the given column are not significantly different, whereas those with different superscript letters are significantly (*p* < 0.05) different as judged by Duncan’s multiple test, df: degree of freedom.

### Apoptosis assay

**Figure 1.**
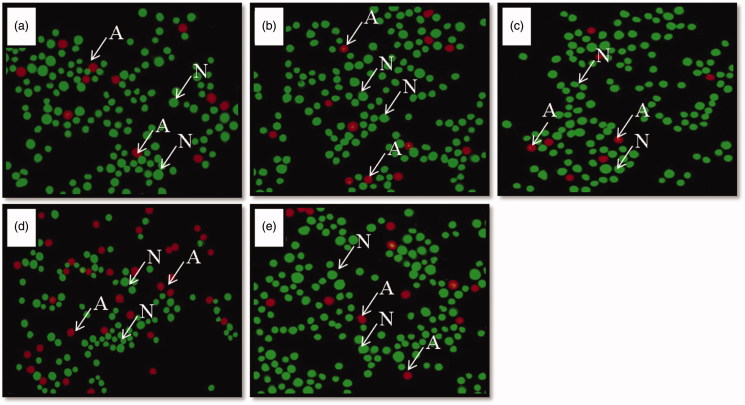
(a–e) Photomicrographs of splenocytes stained with acridine orange and ethidium bromide (450** **nm and 530** **nm). Note the presence of more number of healthy cells (N) in control (a), vehicle control (b), unstressed** **+** **Vacha extract treated rats (c) and stress** **+** **Vacha extract treated rats (e), and more number of apoptotic cells (A) in stressed (d) rats. 200×. A: apoptotic cells; N: normal, healthy cells.

The apoptotic index of cells of the spleen, thymus and axillary lymph nodes was significantly higher in stressed rats compared to controls, whereas, that of Vacha extract pretreated stressed rats did not differ from controls (Table 7). A greater number of healthy cells (green) and less number of apoptotic cells (red) were observed in control (Figure 1(a)), vehicle control (Figure 1(b)), Vacha extract treated unstressed rats (Figure 1(c)) and Vacha extract pretreated stressed rats (Figure 1(e)) compared to stressed rats (Figure 1(d)) in all the lymphoid organs as revealed by fluorescent staining.

**Table 7.** Effects of Vacha rhizome extract on apoptosis of cells in lymphoid organs of rats.

Groups and treatments	Apoptotic index (%)		
	Spleen	Thymus	Axillary lymph nodes
Control	10.600 ± 1.029^b^	12.400 ± 0.927^b^	14.400 ± 1.208^b^
Vehicle Control	11.000 ± 1.581^b^	12.000 ± 1.949^b^	12.800 ± 1.881^b^
Unstressed + benzene extract of Vacha (10 mg/kg body weight)	9.200 ± 0.663^b^	10.800 ± 0.734^b^	11.800 ± 1.067^b^
Stress	25.000 ± 1.843^a^	29.000 ± 2.738^a^	31.200 ± 3.455^a^
Stress + benzene extract of Vacha (10 mg/kg body weight)	11.800 ± 1.593^b^	13.200 ± 1.280^b^	18.400 ± 1.208^b^
ANOVA F Value (df =4, 20)	21.163 *p* < 0.001	20.179 *p* < 0.001	16.149 *p* < 0.001

Mean values with same superscript letters in the given column are not significantly different, whereas those with different superscript letters are significantly (*p* < 0.05) different as judged by Duncan’s multiple test, df: degree of freedom.

### Histology

**Figure 2.**
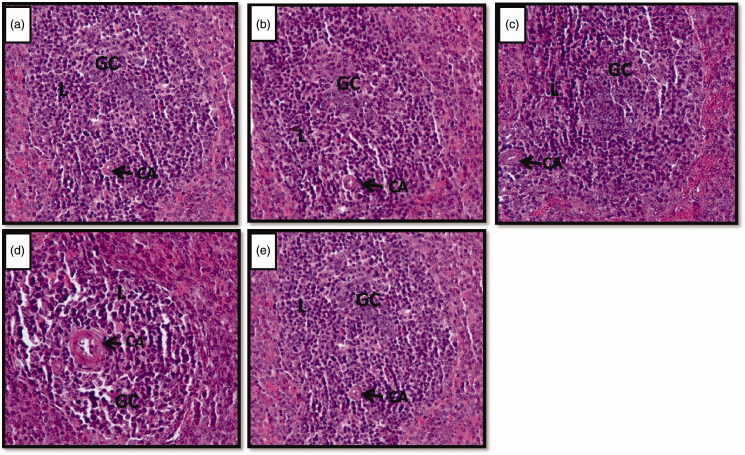
(a–e) Photomicrographs of the cross sections of the spleen showing the island of white pulp. Note the shrinkage of island of the white pulp region and germinal center in stressed rats (d) compared to control (a), vehicle control (b), unstressed** **+** **Vacha extract (c) and stress** **+** **Vacha extract (e) treated rats. 200× (H&E). GC: germinal center; CA: central artery; L: lymphocytes.

A significant decrease in the number of islands of white pulp of the spleen was observed in stressed rats compared to controls. However, in Vacha extract pretreated stressed rats, the number of islands of white pulp did not significantly differ from controls. There was a significant increase in the number of islands of white pulp of Vacha extract treated unstressed rats compared to controls (Table 8). The germinal center and white pulp of control (Figure 2(a)), vehicle control (Figure 2(b)) and Vacha extract treated unstressed rats (Figure 2(c)) did not show variations, whereas, a reduction in germinal centers and shrinkage of white pulp were observed in stressed rats (Figure 2(d)). However, Vacha pretreated stressed rats (Figure 2(e)) resembled controls.

**Table 8.** Effects of Vacha rhizome extract on number of islands of white pulps in spleen of rats.

Groups and treatments	Number of islands of white pulp/cross section of spleen ± SE
Control	27.500 ± 1.322^b^
Vehicle Control	27.250 ± 1.108^b^
Unstressed + benzene extract of Vacha (10 mg/kg body weight)	34.200 ± 1.772^a^
Stress	21.500 ± 1.322^c^
Stress + benzene extract of Vacha (10 mg/kg body weight)	26.600 ± 1.326^b^
ANOVA F Value (df =4, 20)	10.217 *p* < 0.001

Mean values with same superscript letters in the given column are not significantly different, whereas those with different superscript letters are significantly (*p* < 0.05) different as judged by Duncan’s multiple test, df: degree of freedom.

**Figure 3.**
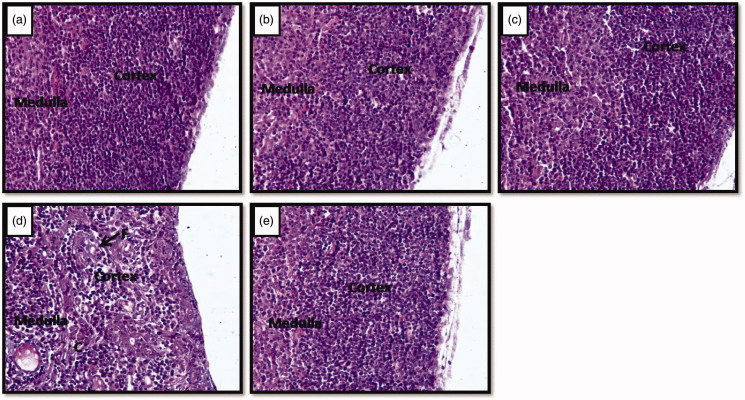
(a–e) Photomicrographs of the cross sections of thymus gland. Note the reduction of cortex region and development of connective tissue and fat cells in stressed (d) rats compared to control (a), vehicle control (b), unstressed** **+** **Vacha extract (c) and stress** **+** **Vacha extract (e) treated rats. 200× (H&E). C: connective tissue fibers; F: fat cells.

The cortex region of the thymus of controls (Figure 3(a)), vehicle control (Figure 3(b)) and Vacha extract treated unstressed rats (Figure 3(c)) did not show histological alterations, whereas it was shrunken in stressed rats (Figure 3(d)). However, Vacha pretreated stressed rats (Figure 3(e)) resembled controls.

**Figure 4.**
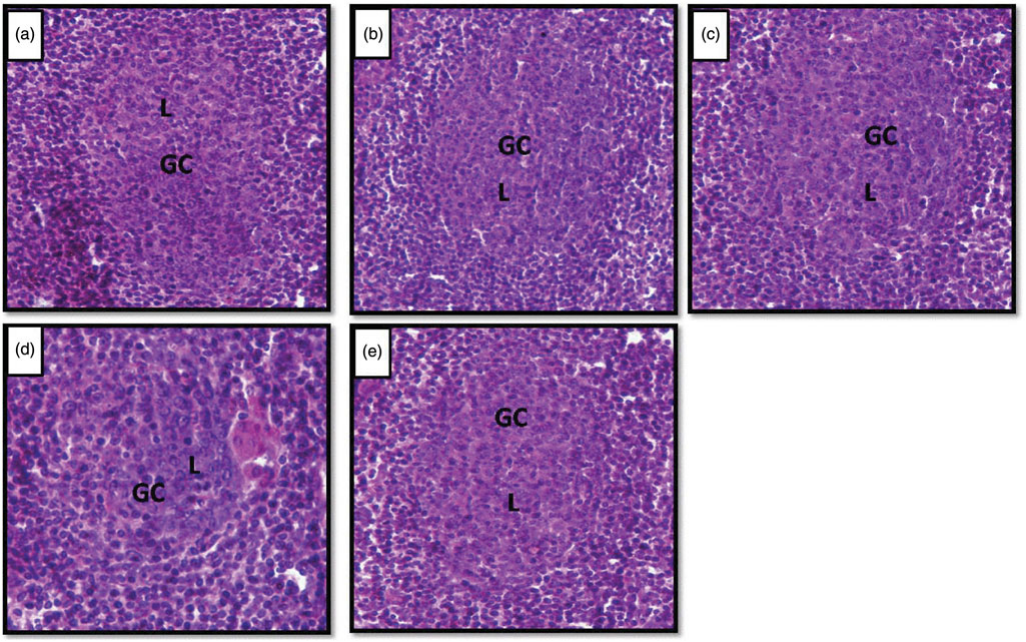
(a–e) Photomicrographs of cross sections of axillary lymph node showing lymphoid follicles. Note the shrinkage of germinal centers in stressed (d) rat compared to control (a), vehicle control (b), unstressed** **+** **Vacha extract (c) and stress** **+** **Vacha extract (e) treated rats. 200× H&E. GC: germinal centre; L: B lymphocytes.

The normal architecture of lymphoid follicles of axillary lymph node was observed in control (Figure 4(a)), vehicle control (Figure 4**(**b)) and Vacha extract treated unstressed rats (Figure 4(c)), whereas, a shrinkage of the germinal center of the lymphoid follicle was observed in stressed rats (Figure 4(d)) but not in Vacha extract pretreated stressed rats (Figure 4(e)).

## Discussion

The present study demonstrates immunoboosting as well as immunoprotective property of a benzene extract of Vacha as shown by significant elevations in all immune parameters in unstressed rats and near normal state in stressed rats following treatment with the extract. It is remarkable that Vacha treatment even in normal rats is beneficial as it enhances immunity.

Immunomodulation is alteration in immune responses which may increase or decrease the immune responsiveness (Mukherjee et al. 2014). The immune response of an organism is altered by the immunomodulators via regulatory mechanisms. These mechanisms may either be antigen dependent or directly induce the production of mediator and effector molecules by the immunocompetent cells (Kumari et al. 2014). Rhizome extract of Vacha proved to be an immunomodulator as it stimulated the immune responses in our present study as shown by significant increases in relative weight of the lymphoid organs, total leukocyte count, differential leukocyte count, immunoglobulin content, bone marrow cellularity and viability, lymphocyte count of lymphoid organs and decrease in circulating immune complexes and apoptosis of lymphocytes in lymphoid organs in Vacha extract treated unstressed rats. The immunomodulatory effect of Vacha is similar to other herbal extracts viz. *Withania somnifera* (Verma et al. 2012), *Picrorhiza kurroa* L. (Plantaginaceae) (Siddiqui et al. 2012) and *Asparagus racemosus* L. (Asparagaceae) (Parameshwara et al. 2015). In contrast to these studies, Mehrotra et al. (2003) reported the immunosuppressive action of ethanolic extract of Vacha on mouse and human cell lines. The increased relative organ weight of lymphoid organs in Vacha treated unstressed rats indictaed the activated immune response by lymphocytes. Total leukocyte count and differential leukocyte counts are earliest indicators of immune responses as leukocytes in the blood are the first cells to respond to immunomodulators (Sumalatha et al. 2012). In the present study, Vacha extract treated unstressed rats showed significantly higher total and differential leukocyte count indicating the initial triggering of blood cells to mount a potent immune response. Increased neutrophil levels in the Vacha treated unstressed rats suggest the antibacterial and antifungal property of the extract as neutrophils constitute the first line of defense (Okon et al. 2011). The common hemapoietic stem cells of bone marrow are the source of major cell types involved in immune system. The present study reveals stimulatory effect of Vacha on bone marrow cellularity and viability of cells and on other lymphoid organs. It is suggested that Vacha extract treatment enhanced the bone marrow cellularity and viability leading to increase in production of blood leukocytes that subsequently resulted in an increase in total blood leukocyte count. In addition, normal apoptotic index, higher lymphocyte count of lymphoid organs, more number of islands of white pulp in spleen, higher proportion of lymphocytes in thymic cortex, larger lymphoid follicles of axillary lymph node, larger germinal center and white pulp in spleen of Vacha rhizome treated unstressed rats compared to controls clearly indicate the stimulatory effect of the Vacha extract on different lymphoid organs. Thus, Vacha extract improves the normal functions of the lymphatic system. The Vacha rhizome extract lowered the circulating immune complex levels compared to controls and thereby indicated an efficient phagocytosis. Escape of circulating immune complexes from phagocytic clearance provokes an inflammatory damage (Parveen et al. 2010) and progression of cancer (Balaram et al. 1987). In addition, there was an increase in total immunoglobulin content after Vacha supplementation which could be attributed to the increase in number of antibody secreting cells. The fact that a dosage of 10 mg/kg bw Vacha rhizome extract, which is lower compared to earlier studies on herbs, significantly simulated the immune responses, reveals a potential immunoboosting property of the Vacha and thus helpful in maintaining disease free state.

Activation of adrenocortical activity is a familiar response of vertebrates to stress (Devaki et al. 2013). Hence, activation of adrenocortical activity in the present study as shown by an increase in 3β-HSDH activity and weight of adrenal gland following exposure to restraint and forced swimming indicates that these rats were experiencing stress and alterations in immune parameters of these rats were due to stress. Several earlier studies have reported stress-induced depletion of blood leukocytes (Moazzam et al. 2013), bone marrow cellularity (Kemeny & Schedlowski 2007), interference with immunoglobulin production (Moazzam et al. 2012, 2013) and reduction in plasma antibody titers (Archana & Namasivayam 2000). Similar to the earlier reports, in the present study, stress alters the cell mediated as well as humoral immunity in rats as shown by a significant decrease in total and differential counts of leukocytes, cellularity and viability of bone marrow cells and total immunoglobulin content of blood in rats exposed to restraint and forced swimming. In addition, circulating immune complexes represent an immunological effector mechanism for antigenic clearance (Contreras et al. 1982). In the present study an increase in circulating immune complexes of blood in stressed rats indicates depletion of antigenic clearance. Thus, this study utilizing different parameters clearly demonstrates that chronic stress adversely affects cell mediated as well as humoral immunity. It is interesting to note that none of these parameters significantly differed from controls in Vacha extract pretreated stressed rats thereby indicating an immunoprotective effect of Vacha. The humoral immunity involves interaction of B lymphocytes with an antigen and subsequent production of antibody secreting plasma cells (Gokhale et al. 2003). In the present study stress induced reduction in blood immunoglobulin content might be due to stress induced reduction in lymphocytes. This view is supported by the facts that normal lymphocyte count in Vacha treated stressed rats was accompanied by normal immunoglobulin content and an increase in lymphocyte count in Vacha extract treated unstressed rats was accompanied by an increase in immunoglobulin content. In addition, Vacha extract also enhances the survival of lymphocytes as is shown by a decrease in apoptotic index of lymphocytes in Vacha extract treated unstressed rats compared to controls and apoptotic index similar to controls in Vacha treated stressed rats. The present study by comparing the effects of Vacha in unstressed and stressed rats reveals for the first time that, Vacha enhances bone marrow cellularity and viability and lymphocyte proliferation and survival, boosts cell mediated as well as humoral immunity in normal rats and exerting similar effects maintains normal immune status in stressed rats.

Immunity of the body is the consummate effect of different lymphoid organs. However, earlier studies focused on immunoprotective property of herbal extracts on blood leukocytes and related mechanisms, whereas primary and secondary lymphoid organs were least investigated. In addition to bone marrow, the present investigation showed shielding action of Vacha rhizome on the spleen, thymus, and axillary lymph nodes under stressful conditions. Stress exposure significantly decreased the relative weight of lymphoid organs compared to controls. However, oral administration of rhizome extract attenuated the stress induced loss of weight of lymphoid organs. This restorative property of Vacha rhizome could be due the prevention of lymphocyte loss in these lymphoid organs under stressful condition as the counts of lymphocytes of lymphoid organs in Vacha extract pretreated stressed rats were similar to controls and there was a reduction in apoptotic index of lymphocytes. Few studies have reported stress induced thymic involution (Tarcic et al. 1998), reduction in the germinal center of lymphoid follicle and loss of islands of white pulp of the spleen and decrease in size of the follicles and the marginal zone of the spleen (Hernandez et al. 2013). Similar to the earlier reports, in the present study chronic stress resulted in shrinkage of white pulp and germinal center and loss of islands of white pulp of the spleen, thymic involution and shrinkage of germinal center of axillary lymph node. In addition, stressed rats exhibited significantly higher apoptotic index in spleen, thymus and axillary lymph nodes compared to controls. Our present study for the first time demonstrates that, Vacha rhizome pretreatment effectively prevents stress induced alterations as Vacha extract treated stressed rats did not significantly differ from controls in any of the parameters studied.

Glucocorticoids are the key mediators of stress responses affecting development and function of the immune system and induces apoptosis of blood leukocytes (Schmidt et al. 1999), bone marrow cells (Rossi et al. 2007), lymphocytes of lymphoid organs (Tarcic et al. 1998) and brings immunodeficiency. In the present study, reduction in leukocyte population of blood, lymphocytes of lymphoid organs, immunoglobulins and increased circulating immune complexes may be due to elevated levels of glucocorticoids. Higher levels of glucocorticoid lead to autrophy of lymphoid organs and suppress both humoral and cell mediated immune mechanisms (Kubera et al. 1998). The significant decrease in the weight of lymphoid organs of the stressed rats in the present study may be the result of increased production of glucocorticoids which in turn lead to the lysis of glucocorticoid sensitive thymocyte, splenocytes and lymphocytes of axillary lymph nodes. In the present investigation, increased adrenal activity as shown by increased adrenal weight and 3β-HSDH activity in stressed rats indicated the activation of the HPA axis which was accompanied by immunosuppression. However, normal adrenal 3β-HSDH activity and weight of adrenal gland in Vacha extract pretreated stressed rats indicated that Vacha extract prevented the stress-induced activation of adrenocortical activity and maintained normal immune status despite exposure to stressors. Hence, it appears that rhizome extract of Vacha alleviates stress induced immunodeficiency by suppressing stress induced activation of the HPA axis. However, the direct influence of the extract on lymphoid organs is also possible. Further studies are needed to confirm theses views.

## Conclusions

The present study clearly demonstrates that stress induces suppression of cell mediated as well as humoral immunity and structural and functional involution of primary and secondary lymphoid organs, whereas the benzene extract of Vacha prevents these alterations in stressed rats. Vacha extract alleviated the stress induced immunodeficiency by suppressing stress-induced activation of HPA axis as indicated by the normal adrenal 3β-HSDH activity and weight of adrenal gland in Vacha extract pretreated stressed rats. Therefore, it is suggested that Vacha extract administration maintains normal immunity despite body experiencing stress.
